# *QuickStats:* Age-Adjusted Death Rates for Males, Females, and Both Sexes — United States, 2009–2018

**DOI:** 10.15585/mmwr.mm6931a5

**Published:** 2020-08-07

**Figure:**
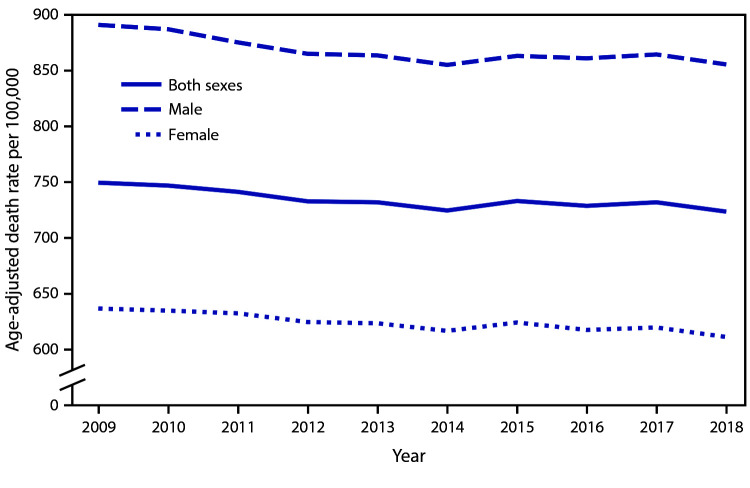
During 2009–2018, the age-adjusted death rate in the United States generally declined, from 749.6 per 100,000 in 2009 to 723.6 in 2018. The death rate among males declined from 2009 (890.9) to 2014 (855.1), increased in 2015 (863.2), and then remained relatively flat until 2018 (855.5). Among females, the death rate declined steadily from 2009 (636.8) to 2018 (611.3). Throughout this period the death rate for males was higher than that for females.

**Source:** National Center for Health Statistics, National Vital Statistics System, mortality data. https://www.cdc.gov/nchs/nvss/deaths.htm.

